# Compositionality beyond bases

**DOI:** 10.1098/rstb.2024.0209

**Published:** 2025-10-20

**Authors:** Jean-Charles Pelland

**Affiliations:** ^1^ University of Bergen, 5015 Bergen, Norway

**Keywords:** numerical cognition, compositionality, numerals, base, unit, recursion

## Abstract

This paper situates bases within a larger family of compositional tools, using a novel conceptual framework centred on numbers used as counting units, which I label *anchors*. I motivate introducing this framework by showing that existing definitions of bases in linguistics and in the study of notation diverge significantly from each other, making neither suitable for a more general discussion of numeration systems across all representational formats. While notational definitions lump too many systems under the ‘not-a-base’ label, linguistics-inspired approaches allow too many systems into the base kingdom. I illustrate the broad range of compositional tools that need to be included in a format-independent discussion of numeration systems by showing how perceptual grouping in tallies instantiates shifts in counting units and how place-value can implement a recursive unit-labelling function. I highlight the advantages of an anchor-based framework by applying it to some well-known lexical systems.

This article is part of the theme issue ‘A solid base for scaling up: the structure of numeration systems’.

## 1. Introduction

The concept of the base of a numeration system has been productively used to analyse and categorize both numerical notations and lexical numerals. However, as I detail below, the way many linguists have framed the concept of a base deviates significantly from how bases are defined in mathematical notations. While it has not necessarily hindered intra-disciplinary progress, this conceptual clash slows inter-disciplinary research on important questions about our numerical abilities, including investigation into their potential origins in body-based numerals.

This article has two main objectives: first, to show that the conflicting way the concept of base has been used in the study of notations and of lexical numerals is not well suited for a general investigation into the compositionality of representations of numbers across representational formats. Second, to lay down the roots of a conceptual framework capable of fulfilling this role. To achieve these goals, the rest of the article unfolds as follows. In §2, I illustrate how bases of mathematical notations are defined using a few familiar examples. I then show that this definition lumps many different systems under the unhelpful ‘not-a-base’ label in §3 by applying it to lexical numeral systems. Section 4 explores how some linguists have defined bases and shows how this approach also has a lumping problem, but under the ‘base’ label. Having shown the limitations of these definitions, §5 then takes us through tallies to pump intuitions about what sort of features may be important to capture in a broad conceptual framework, focusing especially on shifts in counting units. Section 6 then illustrates the value of using counting units as a core concept with which to analyse numeration systems by comparing how Indo-Arabic and Roman numerals individuate these, since only the former does so via a recursive function. This leads to a definition of base in terms of powers of a number as counting units and to the introduction of a broader compositional tool that I call anchors. Finally, §7 introduces the basics of anchors and illustrates their potential as analytical tools by applying them to concrete examples.

## 2. Base basics

Much like we can differentiate between languages based on their lexicon and grammar, to set numeration systems apart from one another, we can track two characteristics of these: (i) their *signary*, which is the inventory of atomic^1^ numerals of a system, and (ii) the compositional rules that operate on the signary. Roughly speaking, the idea here is that if we know what the system’s building blocks are (i.e. the signary) and we know how to combine these building blocks together (using the compositional rules), we have a good idea of how that system allows its users to produce representations of numbers (i.e. numerals).

For example, in simple tally systems, there is one sign (or symbol),^2^ a mark like ‘I’ or ‘•’, and one rule that goes something like ‘add a mark to produce the sign for the next number’. It is easy to describe the elements of the signary of Indo-Arabic numerals (IANs hereafter): these are the familiar numerals ‘0’, ‘1’, ‘2’, ‘3’, ‘4’, ‘5’, ‘6’, ‘7’, ‘8’ and ‘9’.^3^ However, the task is less obvious when it comes to a precise formulation of IANs’ compositional rules. Despite their apparent simplicity to those familiar with them, IANs take a long time to learn and master [4].

One rule that is often mentioned is that the Indo-Arabic system is *positional*: the value of a numeral depends not only on the value of the signs it contains but also on their position. It is also common to hear that the Indo-Arabic system, like many others, is *structured by a base*. Despite some variation, definitions in the literature on notations seem to agree on essential features of what constitutes the base of a notation. Consider, for example, Widom & Schlimm’s [5] definition: ‘a number b whose powers b^0^, b^1^, b^2^ . . . b^n^ receive special significance, often in the form of their own signs, with the effect of collecting all previous lesser units into a new, greater unit’ (p. 158).

Applying this definition to IANs, we see that in this system, it is powers of 10 that receive special significance by getting specific positions attributed to them in a numerical expression. Symbols used in any position other than the rightmost one will track units comprising more than one element—i.e. units containing numbers of elements that are powers of *ten*. So in ‘832’, the ‘8’ tells us we have 8 units worth 100 elements each, 3 units containing 10 elements and 2 singleton units. This is different from, say, Roman numerals, where extracting the value expressed in a numeral phrase is a matter of adding the value of all the atomic numerals that compose it, which only requires knowing the meaning of each sign present in the phrase, irrespective of position.

## 3. Beyond the page: the dirt on bases

While notations have been analysed in great detail (e.g. in [6]), this pales in comparison to the attention lexical numerals around the globe have received. In a colossal body of work spanning hundreds of years, linguists have documented an incredible variety of numeration practices, many of which do not seem to share features of notational bases. Consider for example the lexical numerals of Kala Lagaw Ya, a language found in the Torres Strait (between Australia and New Guinea), where only two atoms make up the signary, and the system’s extent only goes up to 6 [7, p. 105]. Here, 1 is reported to be ‘urapun’, 2 is ‘okosa’, 3 is ‘okosa urapun (i.e. ‘two one’), 4 is ‘okosa okosa’ (i.e. ‘two two’), 5 is ‘okosa okosa urapun’ (‘two two one’) and 6 is ‘okosa okosa okosa’ (i.e. ‘two two two’). For any value above six, the term ‘ras’ is used, meaning ‘a lot’. Making things more complicated is the fact that this lexical system is not completely conventionalized, since 5 can also be labelled ‘urapuni-getal’, meaning ‘one hand’.

Remarking on the limited numerical extent and the lack of conventionalization for expressing 5 of this language, Conant quips that calling this a system gives ‘to the legitimate meaning of the word a severe strain’ [7, pp. 105−6]. Setting aside the question of what counts as a numeration system,^4^ the important point for us here is that Conant’s remark about the illegitimacy of calling this a system could arguably be said for the legitimate meaning of the words ‘base’ and ‘binary’ in this case, especially if we apply the notational definition to it.

On the one hand, given the role 2 plays in generating these lexical numerals, it is certainly tempting to consider this as a binary system, even though the label for 5 can optionally be obtained without operations on 2. And if ‘binary’ means ‘base−2’, then it is tempting to call this a base-2 system, to reflect the important role 2 has for the language’s numeral composition rules. On the other hand, however, it is clear that we are worlds apart from how bases are defined in the study of notation, since there are no special labels for powers of 2. Rather, instead of having a new label equivalent to ‘four’ for 4, we have ‘two two’, and given that there are no labels for 8, we have no special label for that power of 2 at all.

Of course, there is nothing in the notational definition preventing a system from having 2 as its base, as is clearly illustrated by the importance of binary code in the world of computing. Moreover, there are many systems that exploit compositional principles on numbers larger than 2 that would still not be considered bases under the notational approach. Consider, for example, the numeration system of the Maipures of the Orinoco [7, p. 56]:

1. papita. 2. avanume. 3. apekiva. 4. apekipaki. 5. papitaerri capiti = 1 only hand.
6. papita yana pauria capiti purena = 1 of the other hand we take.
10. apanumerri capiti = 2 hands.
11. papita yana kiti purena = 1 of the toes we take.
20. papita camonee = 1 man.
40. avanume camonee = 2 men.
60. apekiva camonee = 3 men, etc.

We see that this system has atomic labels for individual numbers up to 5, but also numerals that label special units of different sizes (5 = hand, 20 = man), from which other numerals can be formed. Despite its larger extent and the use of systematic compositional tools, the available data show no evidence that powers of a number receive special labels here. So, even if we only require that *some* powers of the base have special labels, as Chrisomalis’ [6, p, 4] notational definition proposes, this system would not be considered as having a base.

These are but two examples. There are myriad systems that exploit similar compositional tools across the world’s languages, but also beyond language, including many body-based systems that do not have sufficient extent or compositional tools to have special labels for powers of a number (discussed in [9]).

Yet if we try to apply notational definitions, these are all equally non-base systems.

By lumping together all these numeration practices under a single ‘not-a-base’ label, we lose track of all their differences. This is exactly the opposite of what is desired in a conceptual framework whose purpose is to allow discussion of numeration systems across all representational formats, which is our objective here. After all, notations did not appear out of nothing. They have precursors in other numeration practices, and those other numeration practices themselves likely went through many stages of development, under the influence of Darwinian forces of cultural evolution.^5^ If we wish to track those differences and compare numeration practices through evolutionary stages, we need a definition of base that reflects the specific features of notations but also accommodates the compositional tools used outside of notations. The notational definition does not seem to allow us to do this. In the next section, we explore the extent to which definitions from linguistics have a better chance of being exported to a more general study of numeration practices.

## 4. Promiscuous definitions and base bloat

Now, to see if definitions from linguistics fare better in capturing what a base is across different types of numeration practices, we need to pick one or two. Unfortunately, there is considerable variation among linguists regarding what bases are. Indeed, some linguists working on numerals (e.g. [11,12]) avoid the term altogether. Thankfully, Barlow [13] offers a thorough analysis of the notion of base in linguistics, together with the following broad definition that reflects many linguists’ take on the notion of base in lexical numeral systems:

 Base_LING_: A base is a numerical value that is used systematically in the formation of numerals.

This definition does seem to capture some oft-cited linguists’ definitions of bases. For example, it matches Comrie’s [14] rather permissive definition, ‘that numerical value to which various arithmetic operations are applied’, and it seems to apply to his more arithmetically inclined definition, where a base is ‘the value n such that numeral expressions are constructed according to the pattern xn + y, i.e. some numeral x multiplied by the base plus some other numeral’ [15]. Departing slightly from these takes is Conant, who describes a base as ‘a number from which he makes a fresh start, and to which he refers the next steps in his count’ [7, p. 102].

Despite their slight variation, we can see how these definitions apply to the two cases discussed in the previous section. For the Torres Strait system, we see in which sense we make a fresh start using 2: once we have counted to 2, we add 1 to our numeral (by calling 3 with an expression that can be approximately glossed as ‘two and one’), thereby starting a fresh counting cycle. We can also see how we have a systematic pattern 2*x* + *y* for all numerals, where *x* = 0 and *y* = 1 for 1, *x* = 1 and *y* = 0 for 2 and so on, with multiples of 2 in the *x* position, up until *x* = 3 and *y* = 0 for 6.

We can also see how these definitions apply to the Maipure system’s many compositional tools. Here, once we get to 5 in our count list, we get 6 by adding 1, thereby making a fresh start in a new counting cycle, and another fresh start at the next hand and each foot, until a fresh start of a different order occurs when we start counting in terms of twenties. As for Comrie’s formula, it gets implemented with 5, as seen by the fact that until we reach 20, we have 5*x* + *y*, where *x* is the number of hands or feet and *y* is an atomic numeral, while at 20 we have 20*x* + *y*, where *x* is the number of bodies and *y* can be broken down into a separate application of 5*x* + *y*.

While these definitions easily accommodate such examples, this is to be expected: just like it is unsurprising that the notational definition works well for notations, here too it was precisely for such domain-specific examples that the definitions were devised in the first place. Now, given that these definitions do not impose power-related constraints on which units receive special labels, they are more inclusive. So inclusive in fact that they lump many lexical systems on top of all known notations under the label ‘base’. In this sense, these definitions mirror the problem we had when trying to export the notational definition to other types of numeration practices. The difference is that here, we lump systems under the ‘base’ banner.

Here, the base bloat takes two forms: one is that the number of compositional tools that are considered bases under such loose terminology dramatically increases when compared to how the notational definition slices up the world of numeration practices. But second, we also see that individual systems can have more than one base, which is not the case in notations. Indeed, in a sense, systems like IANs can be seen as having an endless supply of bases under the linguistic approach! After all, each power of 10 is systematically used in producing numerals in this system. Worse, in linguistics, systems can have many bases that are not related by exponentiation and in ways that differ largely from how sub-bases relate to bases in notations [16]. For example, the rural sign language of LSChicano [17] is described as having several bases due to its use of 10, 20 and 50 as compositional tools. Such base plurality is mentioned in many sources (e.g. [18,19]) and is explicitly considered by some as a feature of certain systems: ‘Based systems can be further classified according to the number of bases used in the organization of counting. Thus, there are monobasic, dibasic, tribasic systems, and so on’ [20, p. 44].

In short, we are likely to suffer from base bloat if we try to export such linguistics-inspired definitions to analyse all numeration practices. While such use of the term ‘base’ to describe so many different types of compositional tools *might* be adequate for within-domain discussions,^6^ if we wish to talk about relations between different types of numeration practices, we need to keep track of differences between different ways of forming numerals. This applies to synchronic relations, but also, perhaps more importantly, historical, diachronic relations. If we wish to identify where bases come from and what sort of cognitive and material resources had to be recruited in the development of early numeration systems, we cannot lump all forms of compositionality in numeration systems under the broad concept of base, since that obfuscates any important differences between systems that we wish to track. Unfortunately, given that so many systems would be considered as having a base under an exported linguistics framework, whatever differences might be helpful in explaining early forms of compositional tools in numeration practices that might have led to the emergence of bases will be hidden due to being labelled under a single banner.

One solution to the base bloat we get from the promiscuous approach to bases seen in linguistics could be to adopt a qualified hierarchy of bases, in a manner analogous to Barlow’s typology of linguistic bases [13, §4]. The idea would be to continue to use the concept of base as it has been used in linguistics, but in a qualified manner, so as to better reflect differences between different types of bases. In other words, instead of it being bases all the way down, we slice up the base universe according to the frequency of use of a particular number in a specified range of the system (e.g. always, regularly, sporadically, once), as well as to the arithmetical operation that is applied to a number to form composite numerals (e.g. addition, multiplication, exponentiation). This way, differences between compositional tools are reflected in the qualification applied to the word ‘base’.^7^ Bases that only exploit addition are different from those that exploit both addition and multiplication, and those that exploit exponentiation are different from the previous two, so that we would have additive bases, multiplicative bases, exponential bases and combinations of these (e.g. additive-multiplicative bases). Similarly, systems that consistently and frequently use certain operations would receive a different categorization than those that do so sporadically and irregularly.

Unfortunately, while this certainly looks like a step in the right direction (and we will propose similar constructions below in §7), it will not quite get us to a terminological framework capable of situating bases in relation to other compositional tools that may be important to track when comparing features of numeration practices across representational formats. There are two main problems with such a qualified framework: one concerns the amount of revisionism we are comfortable with, while the other is related to what we leave out when slicing up the compositional landscape using only arithmetical operations and their frequencies of use.

To understand the first problem with this approach, we must realize that, typically, such qualified schemes have as their unqualified, basic option the one with the *fewest* qualifications. For example, coffee tables, dining room tables, round tables and square tables are all types of tables that carve up the table landscape, in which the basic, unqualified concept of table somehow is meant to share all features that the more qualified versions have, whatever those are. However, typically when we speak of a language’s numerals being base-n or especially of a notation being base-n, we mean it to describe bases that fit the stronger criteria applied to notations, which would be better described as exponential bases on this hypothetical framework. That is, ordinary use of the term ‘base’ in the context of numeration, without qualifications, is normally applied to systems that would be considered as being fully fledged bases, with maximum qualifications, such as bases that exploit exponentiation in notational and lexical systems. Thus, while a framework built around qualifications on bases may help deal with the lumping and base bloat of the promiscuous nature of linguistic conceptions of bases, it would require significant revision of ordinary language use (to the extent that ordinary language is used to talk about numeration bases).

The other problem is that even such a qualified use of ‘base’ in terms of arithmetical operations and frequency of use risks glossing over principles that are used in composing numerals. To see how this is a problem, the next two sections illustrate how tracking shifts in counting units can reflect interesting differences between systems that do not seem to be captured by either arithmetical or frequency-related qualifiers.

## 5. Compositionality before bases

In this section, I argue that perceptually grouping tokens of symbols can, in some cases, constitute a compositional rule that cannot be captured by arithmetical operations, which suggests that we need to look for something more general than arithmetical or frequency-related qualifiers to analyse relations between numeration practices. To do this, I use tallies to ground certain intuitions about how units are instantiated. To this end, consider the following tally based representations of the number 28:

T1. IIIIIIIIIIIIIIIIIIIIIIIIIIII

T2. IIIII IIIII IIIII IIIII IIIII III

T3. IIII IIII IIII IIII IIII III

T1 is considered a simple tally because this type of system is said to represent numbers via one-to-one correspondence between the number of tokens of a sign (in this case, ‘I’) and the number represented. Arguably, there is no compositional tool present here, since users of this type of system need only count the total number of tokens to access the represented number, without having to combine numerals in any way. Instead of requiring that users count up 28 individual tokens, T2 and T3 both organize tokens together into perceptually salient *groups*, as part of a perceptual chunking process.^8^ T2 does this by physically separating groups of five tokens from each other, while T3 adds a new sign in the form of a horizontal bar across tokens to make the perceptual grouping even more salient. In both cases, the perceptual grouping facilitates the process of counting tokens, since we now have two units with which to move up the ladder of numerical magnitude: in both T2 and T3, we can count units made up of single tokens, but we can also count units containing five tokens, because of the effect of perceptual grouping. Moreover, instead of having to start counting at 1, we can start counting on at multiples of 5.

Having this larger unit greatly speeds up the counting process by offering different starting points for counting and by giving different sizes of intervals as we move through the landscape of numerical magnitude. In this sense, grouping explicitly singles out a number other than 1 with which users of a system can count—which we will call here a *counting unit*. Counting units are useful beyond tallies, of course: English speakers will tend to count large quantities in terms of tens, or hundreds, rather than twentysevens, say. While any number can be used to count, counting units have special representational status in systems, which is usually manifested via special signs—or, via grouping, as in T2.

While the difference between T1 and T2–T3 may appear quite insignificant on its own—a mere physical re-organization of tokens—it can tell us something about users of the system and the amount of attention they have paid to their tallies. After all, grouped tallies do not emerge willy-nilly: someone had to decide to keep track of a quantity of things using a tally, and someone using a simple tally must have realized at some point that grouped tokens are easier to read. Even if such groupings were not initially voluntarily produced, the fact that this practice survives and is commonly used to this day shows that a user has reflected on the simple tally and found it wanting.^9^ In this sense, differences between T1, T2 and T3 can potentially reflect different stages in the historical development of numeration practices, so those of us who wish to investigate potential origins of numeration practices need a way of tracking these differences—if only to express hypotheses about how practices developed.

Importantly, the compositional role of perceptual grouping (its creation of a new counting unit) does not seem to be captured in either frequency- or arithmetic-based qualifications.

The case of frequency is rather easy to dismiss: both T2 and T3 have identical frequency of groupings, and T1 has no such groupings, so if we wish to capture the difference between these three systems, it does not look like we can appeal to how frequently they resort to grouping tokens.

The case for arithmetical operations is less obvious, although there is a way to describe these tallies that does not seem to require using arithmetical terms. For example, one could simply read the representation of 28 in T2 as ‘five groups three leftovers’ without further needing to decode how many tokens are in five groups. Indeed, at least on the surface, this looks a lot like what happens in lexical systems like English, where we list off the number of tokens of units in decreasing order of magnitude, as in ‘two thousand five hundred fifty-three’. Under this interpretation, there is no need for much arithmetic, certainly not if this means explicitly entertaining a formula like ‘*x*n + *y*’, in mind. After all, the fact that we perceive a group of tokens as a single unit made up of smaller units is not an arithmetical operation, as confirmed by the literature on chunking [23,24, chapter 3].

Now, perceptual grouping does yield a new counting unit that simplifies reading the tally here. Given its role in producing numerals made up of two distinct counting units, perceptual grouping seems like something a qualified categorization of compositional tools would need to capture. Yet we just saw that qualification on arithmetical operations or their frequency would not capture this role. This suggests that the core notion on which we apply qualifications to discuss compositional tools will need to be broader. But what would this notion be?

Well, we just saw that grouping allows shifts in *counting units*, so perhaps this could be a good candidate. Tracking different ways numeration systems have of instantiating shifts in counting units can help flesh out the difference between T2 and T3: while both add counting units to their compositional repertoire, T2 does so merely by perceptual grouping, while T3 does so with both perceptual grouping and cipherization—the introduction of a sign, or cipher (i.e. the horizontal bar).^10^ Moreover, collecting units together to form larger units, which implements a counting unit shift, is part of the definition of the base of a notational system, seen above. This suggests that the notion of counting unit may be a good building block around which to develop a taxonomy of qualified compositional tools. In the next section, to illustrate the potential of a conceptual framework based on counting units, we consider how these can help explain an overlooked difference between Roman numerals and IANs.

## 6. Compositionality, self-reference and recursion

In systems like notations and lexical numerals, the counting units are not individuated perceptually as they are in tallies, but rather via signs. The reader may be perplexed here: how are signs like ‘X’ and ‘10’ labels for *counting* units? As I use the expression here, counting units are those units that have special status due to their being units we use to move up the ladder of numerical magnitude more efficiently—for example, to count up to the target number via units larger than 1, as seen in the previous section. When we learn to count, we learn to label numbers via atomic numerals, but also via composed numerals made up of special units, which we use to form composite numerals that get us through the numerical landscape more efficiently. In this sense, if we wish to single out certain units as being special, we can focus on counting units. So, for example, in Roman numerals, we can count how many Vs, Xs and Is we have to determine which number is expressed, while in lexical systems like English, we are explicitly told how many of each unit—defined here in terms of powers of the base—is present in the numeral.

To see how we can use counting units as a starting point to capture interesting differences between systems, we can look at how labels for these are formed in familiar notations like IANs and Roman numerals. In the case of Roman numerals, if we wish to know what the counting units are, we can simply list them as follows:

 *UnitSign*_*Roman*_: {I, V, X, L, C, D, M}^11^

That is it. No matter how large the number, the largest unit we have to represent it with is M, i.e. 1000. Now, if we try to list units in IANs in the same way, by giving an extensional definition, something different happens:

 *UnitSign*_*IAN*_: {1, 10, 100, 1000, 10000…}

They might not look like much, but those three dots hide the fact that IANs do not have a fixed unit signary^12^ with which we must work to express any numerical value, unlike the Roman numerals. Rather, for IANs, signs for units are determined by a *function* operating on powers of 10, where each unit of the system has its value determined by its position. In fact, if we wish to do away with the ambiguity of those dots, we need to describe the units in IANs using a *recursive* function, like so:

 *UnitSign*_*IAN*_: For position 1: UnitSign_IAN_ = ‘1’; for all other positions *n* > 1, UnitSign_IAN_(*n*) = UnitSign_IAN_(*n* − 1) with an extra ‘0’ to its right.

To see what I mean here by ‘recursive’, consider the factorial function (represented by ‘!’), which is typically used to illustrate recursion in introductory texts. This function yields the result of multiplying all numbers below a specified value together, as follows:

 *Factorial*: 1! = 1; and for any number *n* > 1, *n*!= (*n* − 1)! × *n*

Here, the value of the factorial for a number *n* is determined by its value for the previous number *n* − 1, with the ‘base case’^13^ (i.e. 1) serving as a starting point for its application. The function displays a form of self-referring, not only because the concept being defined is present in its definition, but also because the value the function gives to an input is determined by the same function’s output to the previous value. Like a serpent eating its tail, the function feeds its output values back to itself.

Taking this back to Roman numerals, there is arguably self-reference in the unit-building rules here too, in that each unit is defined in terms of its predecessor: ‘V’ means ‘five Is’, ‘X’ is ‘two Vs’ and so on, each unit being defined in terms of another in the same system. However, this self-reference is not part of a recursive *function*, since units are given to us in a fixed unit signary. This means we cannot produce numerals for arbitrarily large numbers with the units we are given, without significant perceptual confusion for large numbers, due to the number of Ms involved. This fails to satisfy an important aspect of recursion that ‘recursive functions take their own past output as their next input, and potentially allow (in the absence of processing limitations) indefinitely complex output from even the simplest of functions. This basic idea—a simple route to boundlessly complex computation—is the aspect of recursion that binds all uses of this term together’ [25, p. 75]. We do not have this boundlessness in the case of unit formation in Roman numerals.

We *do* have recursion in the unit-building rules of IANs, however, since here to replace those three dots we need to use a function that refers to itself, as seen in the fact that the definition of UnitSign_IAN_ contains UnitSign_IAN_ itself, to allow units to take on arbitrarily large values. Moreover, just like in the factorial example, the value of each unit sign in IANs is determined in relation to the value of the previous sign (here, by multiplication by 10), all the way to the base case.

In sum, our foray into the maze of recursion illustrates another way that looking at numeration systems in terms of how they instantiate counting units can yield interesting differences between them. In the previous section, a difference between T1 and T2 was explained in terms of how perceptual grouping instantiated a shift in the counting unit. Here, the difference in ease of extendibility between IANs and Roman numerals can be explained in terms of the presence of a recursive rule for labelling counting units. Given the broad range of features that appear to be covered by the notion of a counting unit, it is tempting to see if we can develop a useful categorization scheme around it. Now, seeing as values for counting units in both IANs and Roman numerals are determined by the value of the powers of the base (and a divisor of the base for Roman numerals), it is tempting to conclude that powers of the base determine shifts in counting units, in that new counting units are produced at each power of the base. This suggests we can define bases as follows:

  *Base*: The base of a numeration system is the smallest number whose powers are used as conventionalized counting units, as manifested in the use of special representational vehicles.

Armed with this definition, the important question is whether we can adapt it to obtain a starting point for analysing *all* compositional tools, including those that are not bases but are used in forming composite numerals, such as perceptual grouping. After all, our objective is to have a conceptual scheme that is broad enough to capture relations between numeration systems across *all* representational formats in a way that avoids lumping them together. Given that our definition of base specifically singles out numbers whose *powers* individuate counting units, we can simply strip away mention of powers to talk about compositional tools more generally. Of course, talking about such compositional tools as ‘numbers conventionally used as counting units’ is quite a mouthful, so a more manageable term is required. Here, I propose the use of the neutral term ‘anchor’:

  *Anchor*: An anchor is a number conventionally used as a counting unit, either via an arithmetical operation or other compositional operation, as manifested in the use of special labels.

In a sense, this definition can be seen as a compromise between the definitions of base we covered earlier: if we strip away the mention of powers from the notational definition, we get a focus on units that looks something like ‘a number that collects all previous lesser units into a new, greater unit’. However, this would still be too systematic and regular to apply to the messy, irregular way in which units are constructed in some non-notational numeration systems. At the same time, if we strip away parts of the definitions offered in linguistics, we can remove the systematicity of the broad definition offered by Barlow [13], to get ‘a numeral value used in the formation of numerals’, which gets us quite close to Comrie’s [14] definition of base as ‘that numerical value to which various arithmetic operations are applied’. That was too permissive to be useful as a broad definition of *bases*, but it can be useful in talking about a larger family of compositional tools, if combined with the less arithmetically oriented notion of counting unit, as we have done in our definition of anchor.

Unlike bases, anchors have much more freedom in terms of how they can behave. They do not have to be used consistently, nor regularly, nor even more than once. All that is required for a number to behave like an anchor is that it is used as a counting unit. How it achieves this, how frequently or regularly it does this, are all qualifications that individuate different types of anchors. To get a better idea of what we are talking about, in the next section, we take a look at a few examples of counting units and the types of anchors that they are associated with.

## 7. Carving number beyond its joints: how anchors work

There are many ways to individuate counting units, most of which are associated with a different arithmetical operation.^14^ First, we can form a new counting unit by changing the unit *from which* we start counting. When this happens, we (freshly, as Conant put it) start counting on from a number other than 1. This is instantiated in *additive* anchors. As an example of a purely additive anchor, recall that above we mentioned a language from the Torres Strait where the label for 4 was ‘two-two’, while the label for 3 was ‘two-one’. We see that 2 here is a purely additive anchor since it is used as a starting point for a new counting cycle, where we start at 2 and then add other values.

On the other hand, we also see shifts in counting units when we change the unit *with which* we count. This indicates the behaviour of a *multiplicative* anchor. This is because when multiplying, the number being multiplied determines the size of the steps we take to move up in the scale of numerical magnitude to arrive at a number. So in English, we form decades with multiplication on 10, as in ‘twenty’, ‘thirty’, etc., with the ‘ty’ indicating multiplication by 10. Here, the expressions for 20, 30, 40, etc. are all obtained solely by multiplying 10, so these numerals are examples of the use of a purely multiplicative anchor of 10.

Systems that only have multiplicative anchors are very rare, but they do exist, according to Greenberg [1], who mentions a single case of a system that only has numerals for numbers 1−10 where multiplication is the sole operation used to compose numerals: ‘the YUMAN languages…where the numerals may be analysed as follows: 1, 2, 3, 4, 5, 3×2, 7, 4×2, 3×3, 10’ (p. 258). We see here that 2 is multiplied by 3 and by 4, and in both cases there is a discontinuity in counting units, since we were counting in intervals of 1, but then shifted to intervals of 2 to compose numerals for 6 and 8. The label for 9, expressed here by 3 × 3, shows another counting unit shift, since we are moving from using 2 as the unit with which we count to 3.

In each of these cases, the use of multiplication determines the number expressed without any other operation being applied. Typically, however, a number that serves as a multiplicative anchor will serve *both* as an additive anchor and a multiplicative anchor, or as what we will call a *multiplicative-additive* anchor. In such cases, the result of the multiplication serves as the basis to start a new counting cycle, so we have two shifts in units, one directed by the multiplication and the other by the addition on the result of the multiplication. Thus, in English, we see 10 used as a multiplicative-additive anchor in all numerals representing numbers between decades apart from the teens, where it is used as a purely additive anchor. Changing values of the multiplier for other decades yields a new shift in the counting unit, in that the multiplication operation determines a new starting point to which addition is applied. For example, in the English expression of 98, 10 is used as an additive-multiplicative anchor since it is 10 that is used as a counting unit 9 times, and it is on the result of *this* operation on 10 that we start counting to 8, in order to get to 98. In this sense, while the starting point of the new counting cycle is 90, we reflect the fact that we get to our starting point of 90 via multiplication by considering 10 to be a multiplicative-additive anchor.

Sure, technically, we can consider 90 an additive anchor here, since it is the number at which we start a new counting cycle. However, there are two reasons it is better to interpret cases like 98 as the result of the application of a multiplicative-additive anchor. First, labelling all new starting points obtained via multiplication as additive anchors, while technically correct, does not reflect the role of multiplication in setting all these starting points to additive operations. For purely additive anchors, addition is the only operation involved in the composite numeral, as the examples above illustrated. This difference should be reflected somewhere. Second, and more importantly, considering 90 as an additive anchor faces us with an anchor bloat problem, since the result of every use of a multiplicative anchor outside of whole numbers would have to be considered a separate additive anchor, so that characterizing a language like English in terms of its anchors would involve a ridiculously large number of additive anchors, one for each time 10 is multiplied by another number. Considering 10 to be a multiplicative-additive anchor here solves both these categorization problems. Thus, to avoid ambiguity, we should only consider anchors as being *purely* additive when no other operation was used in order to arrive at the augend (i.e. the number to which addition is applied). Whenever another operation is used, we should mention it when categorizing the anchor. In this sense, there are no purely additive anchors in English, apart from 10 itself in the teens range, since any other additive anchor is best thought of as a multiplicative-additive anchor or an exponential-additive anchor.

In English, we see one anchor, 10, playing many roles (i.e. additive, multiplicative and exponential anchor, as well as their combination). But in some cases, many anchors operate in the same system. Recall the system mentioned above of the Maipures of the Orinoco [7], where we saw traces of counting via body parts in the lexical numerals. Here, 5 is used as a multiplicative-additive anchor in the 5−19 range, while for 20 and beyond, we have use of both 20 and 5 as multiplicative-additive anchors, since we form numerals first by counting the number of men (20s), which indicates use of a multiplicative anchor for 20, and then we *add* the number of appendages (5s), which we obtain by multiplication by 0−3, to the number of men, attributing the role of multiplicative-additive anchor to 20. Then, finally, we add the remaining number expressed by an atomic label for 1−4 to the number of appendages, making 5 a multiplicative-additive anchor as well.

Things get more complicated when a system has an exponential anchor—i.e. a base—since the same number can be used to individuate many counting units, via exponentiation, addition and multiplication. Importantly, each operation can be applied to the same number many times at different processing stages in the formation of a single numeral, which means that in order to reflect the nested structure of the operations^15^ used to arrive at the target number, we need to resort to orders of operation of anchors. There is a lot to unpack here, so let us clarify things a bit with an analogy.

Numerals can be seen as measuring units for numerical magnitude [27]: when we represent a number, we often do so using many units, and we can count how many units of a certain size are required to represent that magnitude. The larger units are like a highway that gets us to the area close to the destination, while the smaller units are like footpaths that get us to the front porch. If we give directions to someone on how to reach a number, we can certainly tell them to keep adding 1s until they reach the desired number, but this is akin to telling someone to start walking until they reach Albuquerque: they might reach their destination, but it will take a needlessly long time and effort, to the point of making the journey unfeasible.^16^ The use of an additive anchor has us moving with unary steps, and we can only start from the value of the anchor, so purely additive anchors are rather limited for long distances. The use of a multiplicative anchor allows us to use larger steps, but without combining it with an additive anchor, we might step over any destinations that are not multiples of the anchor, or fall short, as we are fixed in that size of step. So multiplicative-additive anchors are a definite improvement.

However, if we want to give directions to go to any place whatsoever and do this as quickly and efficiently as possible, we need to be able to use many measuring units, from centimetres all the way to kilometres and light-years and everything in between, and we need to be able to start from anywhere. One way to do this is to use many multiplicative and additive anchors separately, but this can become quite cognitively taxing, given the number of different anchors needed to accommodate numbers of any size. This is where bases, qua exponential anchors, come in handy: if we want to move up and down the number line with any degree of efficiency, we need to have a variety of counting units, and we need to pack that variety in as small a cognitive load as possible. Exponential anchors tell us the lay of the land by giving us both starting points and measuring units of all sizes packed under one type of operation on a single number. The special labels for powers of the base give us all the numerical measuring units we need, which we use with or without multiplication to arrive at augends to which we can then add other values determined by other powers of the base. All this is done in a regular and systematic way, thereby minimizing cognitive load when compared to irregular or pluralistic systems.

Importantly, when we follow directions, we have to do this in a set order, piece by piece, and we cannot complete all steps at once. The same applies to using directions to get to numbers: we follow each operational step separately, typically starting from the largest units and moving our way down to the smaller ones [28]. This is shamelessly exploited by the practice of advertising prices in terms of the smallest possible large unit, combined with the largest possible smallest units, as in ‘$7999’. The fact that we process numerals in stages means that our brain is tricked into associating this price with 7000, even though it is only 1 off from 8000, since the first unit we process, 1000, is multiplied by 7, not 8, and the parsed processing means that the lower value is kept in mind separately from others, thereby tricking us into thinking it is representative of the price.

Such parsed processing determines how we break down complex composite numerals into constituent parts, each of which may recruit different anchors that are then combined with other anchors at different processing stages. We see this parsed processing in Widom & Schlimm’s [5] abstract form of the numerical value expressed by a numeral containing counting units for three powers of a base as follows: m_₃_b² + m_₂_b¹ + m_₁_b⁰. Here, the value of the number is obtained by adding the value obtained by the multiplication (*m_n_*) applied to each power of the base (*b*_*n*_). Each such multiplier–power pair is processed separately, and then the total value is obtained by adding each of these. At each power, we have a new starting point and a new size of step added to our arsenal of counting units.

For example, in the English expression ‘one hundred and seventy-nine’, we use 10 as an exponential-multiplicative-additive anchor, because it is used first as an exponential-additive anchor at the first processing stage to get ‘one hundred’, but then also as a multiplicative-additive one in the second stage of the parsed processing process. It is not a purely exponential anchor since pure exponentiation only leads us to numerals for powers of the base, like ‘million’ or ‘hundred’, while in this case we are adding something to 100. As was the case for multiplication, as soon as we leave these whole values, we need to exploit other compositional principles, either by adding other anchors or adding an operation to the same anchor. If the number had been ‘three hundred and seventy-nine’, 10 would first have been used as an exponential-*multiplicative*-additive anchor, since we would first have reached 100 by exponentiation, and then 300 by multiplication, before adding the value of the next power to this value. So, here we start by using 10 as an exponential-additive anchor to get to 100 as a counting unit, and then in a separate operation, we use 10 as a multiplicative anchor to get to 70, since we take steps of decimal size seven times, after which we use 70 as an additive anchor to which we add 9. Because 10 here gets us to the starting point of a new counting routine (70) via multiplication, it is a multiplicative-additive anchor. Adding another power adds another processing stage and another separate use of an anchor. For example, ‘three thousand one hundred and seventy-nine’ means that we are using 10 as an exponential-multiplicative-additive anchor, since we use exponentiation and multiplication to reach 3000, to which we add the remaining numbers obtained by following the processing stages we just went through.

Such breaking numerals down into their constituent anchors means that we can reflect cognitive differences displayed in different ways of expressing the same number within the same system. For example, saying ‘twenty-four hundred’ is common in English, though the more formally correct way of expressing this is ‘two thousand four hundred’. In the first version, we use 10 as a purely multiplicative anchor, counting 24 times 100 in a single processing stage, while in the second version, we have 10 as an exponential-multiplicative anchor (with exponential value at 3 and multiplication value at 2) at one stage, and then 10 is used again to determine a shift in counting unit shift as an exponential-multiplicative anchor at a separate processing stage, with exponential value of 2 and multiplicative value of 4. These presentational differences of the same number have cognitive ramifications, in that they will influence how we process numerical information, as seen in syntactic priming effects (e.g. [29]) and in cross-format influences of numeral syntax. For example, the use of different syntactic structures for spoken lexical numerals will influence how they are transcribed in handwritten format [30].

We can also express anomalies in systems in terms of their anchors. For example, we can break down the French expression of 179, ‘cent soixante dix-neuf’, which can be glossed as ‘hundred sixty ten nine’. In the 70−99 range, French gives us bad directions, since they take us to the right place, but in an inefficient way: instead of taking the multiplicative-additive highway until exit 7, we exit at 6 and then use 10 separately as a purely additive anchor on 60 to express numbers in the 71−79 range. So 10 is used as a multiplicative-additive anchor to get to 60, but instead of adding a sub-10 value to it as has been done in other decades, the input to the addition on 60 is the use of 10 as a nested purely additive anchor to reach 19. This is then added as input to the use of 10 as a multiplicative-additive anchor that got us to 60. As if this was not bad enough, French gets worse in the 80−100 range. Here, French uses 20 as a multiplicative-additive anchor for the 80−90 range, before combining this with 10 as a purely additive anchor for the 91−99 range. So we are not only shifting counting units, but we are also doing this in a way that is normally used to combine values *between* powers in a parsed and nested way. Bad directions indeed!

## 8. Conclusion

I started thisarticle by discussing a strong divergence between how the concept of base is implemented in linguistics and in numerical notations and how each of these approaches was difficult to use as a format-independent definition of bases. I then illustrated how counting units could be an interesting starting point for such a definition by looking at perceptually instantiated differences in counting units in tallies and differences in sign-producing rules for counting units in Roman numerals and IANs. This led me to introduce anchors as numbers that implement new counting units across numeral formats, which I illustrated via a few examples in the last section.

From the few examples we have looked at, we can see how we might use anchors to characterize numeration practices and analyse relations between these. However, the anchor-centric framework proposed here is new and untested. This means there are still open questions, for example, regarding which features we wish to track—does a system have a main anchor, a lowest one, a highest one, how many, are any of them a sub-base, etc. Answering such questions will likely mean revisions to the framework, a few categorization headaches, but also, ideally, revisions in how we see bases and other compositional tools involved in representing numbers, to accommodate input from many disciplines. Indeed, hopefully the framework proposed here will show its value not only in helping categorize different numeration practices under a single unifying conceptual framework, but also help more general reflection on the practice of arithmetic as a whole.

## Data Availability

This article has no additional data.

## Footnotes

^1^
‘Atomic’ here is meant to emphasize the fact that some numerals cannot be decomposed into parts that themselves refer to numbers. The term ‘atomic’ is used here both because it is used in an analogous way in discussions of lexical numerals [1], but also because, much like atoms, which are made of quarks and other smaller particles, atomic numerals can also be made up of other types of entities (e.g. letters), though these lower-order entities do not themselves represent numbers. Non-atomic (i.e. composite) numerals are constructed from more than one atomic numeral. For example, in English, ‘twenty-one’ has three atoms, each of which has numerical content: ‘twen’, ‘ty’ and ‘one’. In a sense, this is a more general version of ‘numemes’ [2], which are the smallest constituents of number words with numerical meaning. For more elaborate treatment of this and other central terms, consult the Glossary attached to this special issue [3].

^2^
Throughout this article, I use the terms ‘sign’ and ‘symbol’ interchangeably, in a non-technical sense, to refer to any material form that is conventionally used as a representation. Signs for numbers—aka numerals—include words, phrases, morphemes, shapes drawn on a piece of paper or lit up on a screen, knots, pebbles, notches on a bone, gestures and anything else that is used to refer to a number.

^3^
I use IANs and English words without quotes to talk about numbers. When these are in quotes, it is to talk about the sign itself.

^4^
While there may be legitimate reasons to be reluctant to talk about such numeration practices as *systems*, or as having bases, one must be careful not to adopt colonial attitudes towards differences between modern western numeration practices and others, as Conant seems to have done in this survey from the late 1800s. See [8].

^5^
In *The Descent of Man*, Darwin noted an analogy between the evolution of species and the evolution of language. Similarly, Chrisomalis [10] notes that a useful way of thinking about numeration systems is to see them as species.

^6^
It is worth noting that base pluralism can lead to confusion when it comes to using words like ‘binary’ or ‘decimal’ to apply to systems, as illustrated by Conant [7, p. 112] describing an otherwise decimal system as containing a ‘binary-ternary-quaternary vagary’ to reflect the fact that other numbers play compositional roles.

^7^
An example of this is Hanke [21, pp. 68−69], who distinguishes between additive bases and multiplicative bases.

^8^
As Gobet *et al*. [22] point out, over the years, chunking has come to mean many different things, being applied to both involuntary and voluntary processes of compressing data into discrete units in short-term memory, as well as perceptual grouping of sensory data into subjectively meaningful units as part of a Gestalt-like process. Here, I intend this latter meaning.

^9^
It is difficult to determine whether or not such groupings need to be considered as signs on their own, with the same status as other members of the signary. While this is a substantive matter, thankfully, we need not settle it here, since their status as separate signs does not seem required to consider them as compositional tools, since it is not controversial that they are chunked into a single cognitive unit and that we can count this type of unit in the same way as regular tokens. A similar question arises in the context of determining whether or not such grouped signs have special status in some notational systems [5, pp. 174−175].

^10^
Schlimm’s [16] abacus representation of the structure of numeration systems reflects the important foundational role of grouping and cypherization. See especially section 2b in [16].

^11^
There were other signs in use at different times and in different versions of the Roman numerals, including signs for 50 000 and 100 000, as well as the *vinculum*, a bar drawn on top of numerals to signify multiplication by 1000, but their use was not conventionalized to the same extent as the units listed above [6, pp. 109−127].

^12^
The unit signary is the inventory of numerals for counting units. Unlike the system’s signary, which only contains atomic numerals, the unit signary can include composite numerals as well.

^13^
The expression ‘base case’ cannot be avoided, as it is the technical term used to refer to the first application of the function, on which all other applications are based. The word ‘base’ here does not refer to the base of a numeration system.

^14^
While anchors can be associated with subtraction and division, Greenberg’s [1] and Chrisomalis’ [6] work show that, in reality, subtractive numerals are relatively rare, while divisive ones are essentially non-existent, save for a few cases of multiplication by a fraction. In what follows, I simplify the discussion and focus on the more frequent operations of addition, multiplication and exponentiation.

^15^
For evidence that we process numerals via parsed, nested structures, see Dotan & Brutmann [26].

^16^
Unless, for some reason, they live close to Albuquerque.
